# Epidemiology of Shoe Wearing Patterns Over Time in Older Women: Associations With Foot Pain and Hallux Valgus

**DOI:** 10.1093/gerona/glw004

**Published:** 2016-02-01

**Authors:** Hylton B. Menz, Edward Roddy, Michelle Marshall, Martin J. Thomas, Trishna Rathod, George M. Peat, Peter R. Croft

**Affiliations:** ^1^Arthritis Research UK Primary Care Centre, Research Institute for Primary Care and Health Sciences, Keele University, Staffordshire, UK.; ^2^School of Allied Health, College of Science, Health and Engineering, La Trobe University, Bundoora, Victoria, Australia.

**Keywords:** Epidemiology, Hallux valgus, Foot pain, Footwear

## Abstract

**Background:**

Foot problems are prevalent in older women and are thought to be associated with footwear. This study examined women’s shoe wearing patterns over time and evaluated associations between footwear characteristics and foot pain and hallux valgus.

**Methods:**

Women aged 50–89 years (*n* = 2,627) completed a survey that included drawings of four toe-box shapes and four heel heights. For each life decade, participants indicated which footwear style they wore most of the time. Foot pain in the past 12 months and hallux valgus were documented by self-report. Logistic regression examined associations between heel height, toe-box shape, foot pain and hallux valgus.

**Results:**

Wearing shoes with a high heel and very narrow toe box between the ages of 20 and 29 was common, but decreased to less than 10% by the age of 40. Compared with women who had worn shoes with a very wide toe box, the likelihood of hallux valgus increased in those who had worn shoes with a wide (odds ratio [OR] 1.96, 95% CI 1.03–3.71), narrow (2.39, 1.29–4.42) and very narrow (2.70, 1.46–5.00) toe box between the ages of 20 and 29 and those who wore shoes with a very narrow toe box (1.93, 1.10–3.39) between the ages of 30 and 39.

**Conclusions:**

Women wear shoes with a lower heel and broader toe box as they age. Wearing constrictive footwear between the ages of 20 and 39 may be critical for developing hallux valgus in later life.

Footwear plays an important role in protecting the foot. The earliest known footwear, dating from approximately 7,500 BCE, were simple sandals secured around the ankle with rope (1), whereas the first direct evidence of footwear covering the foot dates back to 3,500 BCE (2). The functional role of footwear was diminished by fashion influences from the 15th century, as features such as high heels and extended toe boxes became popular (3). Modern footwear has retained vestiges of these early fashion influences, many of which are thought to be associated with foot pain and deformity (4). Consequently, shoe-related foot disorders represent an important public health problem (5). Indeed, the annual economic impact of foot problems caused by footwear has been estimated as $3 billion in the United States alone (6).

Some styles of women’s footwear encompass two key design features, an elevated heel and a constrictive toe box, that are considered to be particularly detrimental to the foot (7). Biomechanical studies have revealed that heel elevation increases the pressures under the metatarsal heads (8), limits motion of the first metatarsophalangeal joint (9), and increases the stiffness of the Achilles tendon (10), whereas shoes with a narrow toe box increase pressures on the medial side of the foot and between the toes (11). Over time, these changes may contribute to the development of foot pain and deformity. Cross-sectional studies have shown that wearing shoes with an elevated heel is associated with hallux valgus and plantar calluses (12), and wearing shoes with a constrictive toe box is associated with hallux valgus (12,13) and foot pain (12,14). Furthermore, studies involving recall of previous footwear use have reported a protective effect of ‘good’ shoes in relation to foot pain (15) and an association between high heel use and hallux valgus (5,16).

Establishing an association between footwear characteristics and foot problems, however, is inherently difficult, as shoe wearing behaviors are likely to vary as a function of both age and period—the interaction of these producing cohort effects (17). Footwear selection in women reflects changes across the life course in fashion consciousness, identity and health status (18). Furthermore, these age- and life-stage changes in footwear selection are expressed in the context of changing footwear fashions, and their availability and affordability. Therefore, in order to better understand the relationship between footwear and foot problems, it is necessary to adopt a life-course approach to estimate past exposure to potentially detrimental footwear behaviors. Accordingly, the aims of this study were to (i) examine shoe wearing patterns over time in older women in relation to the height of the heel and the shape of the toe box and (ii) evaluate associations between these footwear characteristics and current foot pain and hallux valgus.

## Methods

### Study Design

The design was an unmatched population-based case-control study using prevalent cases, derived from baseline data from the Clinical Assessment Study of the Foot (19). Adults aged 50 years and older registered with four general practices were invited to take part in the study, irrespective of consultation for foot pain or problems. Ethical approval was obtained from Coventry Research Ethics Committee (reference number: 10/H1210/5).

### Health Survey Questionnaire

**Figure 1.**
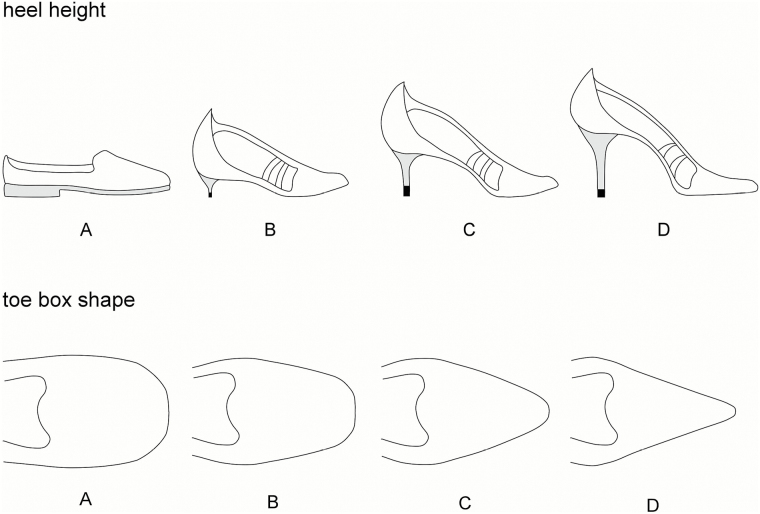
Line drawings depicting heel heights and toe-box shapes.

All eligible participants were mailed a Health Survey questionnaire that gathered information on current demographic and socioeconomic characteristics and general health (19). Past footwear use was assessed by line drawings depicting four heel heights (A = flat, B = low, C = medium, and D = high) and four toe-box shapes (A = very wide, B = wide, C = narrow, and D = very narrow) (Figure 1). For each period of their life (divided into decades, commencing with 20–29 years of age), participants were asked to indicate which heel height and toe-box shape they wore most of the time. Foot pain was assessed with the question “Have you had any pain in the last year in or around the foot?” The presence and severity of hallux valgus was documented using a validated line-drawing instrument, with five drawings illustrating a sequential 15-degree increase in the hallux valgus angle (20). Participants were asked to select for each foot the drawing that best depicted the severity of hallux valgus in that foot. The score was dichotomized for each foot by classifying the three most severe grades as present and the two least severe grades as absent, and participants were deemed to have hallux valgus if this was present on one or both feet (21).

### Statistical Analysis

All analyses were conducted using SPSS Version 21 (IBM Corporation, Armonk, NY). Four birth cohorts were created according to year of birth: 1922–1931, 1932–1941, 1942–1951, and 1952–1961. The frequency and proportion of women who reported wearing shoes with high heels and narrow toe box for each decade of their life according to birth cohort were examined. Associations of past footwear use (heel height and toe-box shape worn when participants were aged between 20 and 29 years, and 30 and 39 years) with foot pain in the last year and hallux valgus were examined using logistic regression, with a flat heel and a very wide toe box as the reference categories. To evaluate the effect of cumulative exposure, the association of the total number of decades of wearing high heels or a narrow toe box with foot pain in the last year and hallux valgus were also calculated. Analyses were stratified by age and adjusted for educational attainment as an indicator of socioeconomic status. Additional adjustment for current body mass index (BMI) was considered, as some (but not all) cross-sectional studies have reported inverse associations between BMI and hallux valgus (16,22,23). However, this was rejected on *a priori* grounds for two reasons. First, adjusting for BMI in our analysis assumes that the BMI documented when completing the Health Survey is a valid measure of each participant’s BMI throughout their adult life. We cannot be certain of this, given the timeframe involved. Second, there is no evidence that BMI influences the selection of footwear. Odds ratios (OR) and 95% confidence intervals (CI) are reported. To estimate the number of cases of foot pain or hallux valgus that could potentially be avoided if footwear risk factors were eliminated, we calculated population attributable fractions (PAF) where significant associations were identified. This involved converting the adjusted ORs to relative risks and calculation of PAF using the formula:

$$\text{PAF}=\frac{\left(\text{aRR}-1\right)}{\text{aRR}}$$

## Results

### Study Population

The baseline Health Survey questionnaire was initially sent to 9,334 adults aged ≥50 years. During the mailing process, there were 140 exclusions due to deaths, ill health, departures, and incorrect addresses. The exclusions left an eligible baseline-mailed population of 9,194 people from whom 5,109 completed Health Survey questionnaires were received (adjusted response 56%) (24). For this analysis, we excluded all men (*n* = 2,439) and due to the small number, women aged 90 years and over (*n* = 43), resulting in a sample of 2,627 women aged 50–89 years. Characteristics of the sample are reported in Table 1.

**Table 1.** Participant Characteristics

Age, mean (*SD*), y	65.6 (9.9)
Body mass index, mean (*SD*) kg/cm^2^	27.6 (5.7)
Higher education attainment	
Yes	466 (17.7)
No	1,994 (75.9)
Missing	167 (6.4)
Employment status (current)	
Employed	630 (24.0)
Retired	1,525 (58.1)
Unable due to illness	124 (4.7)
Unemployed	21 (0.8)
Housewife	178 (6.8)
Other	56 (2.1)
Missing	93 (3.5)
Occupational class (current or previous)	
Managerial/administrative/professional	424 (16.1)
Intermediate	492 (18.7)
Routine/manual	1,355 (51.6)
Other*	356 (13.6)
Foot problems	
Foot pain in the past 12 mo	1,185 (45.1)
Hallux valgus	1,172 (44.6)

*Notes:* Values are *n* (%) unless otherwise noted.

*Includes housewives, nonworkers, retired people and those inadequately described.

### Footwear Characteristics According to Decade of Life and Birth Cohort

**Figure 2.**
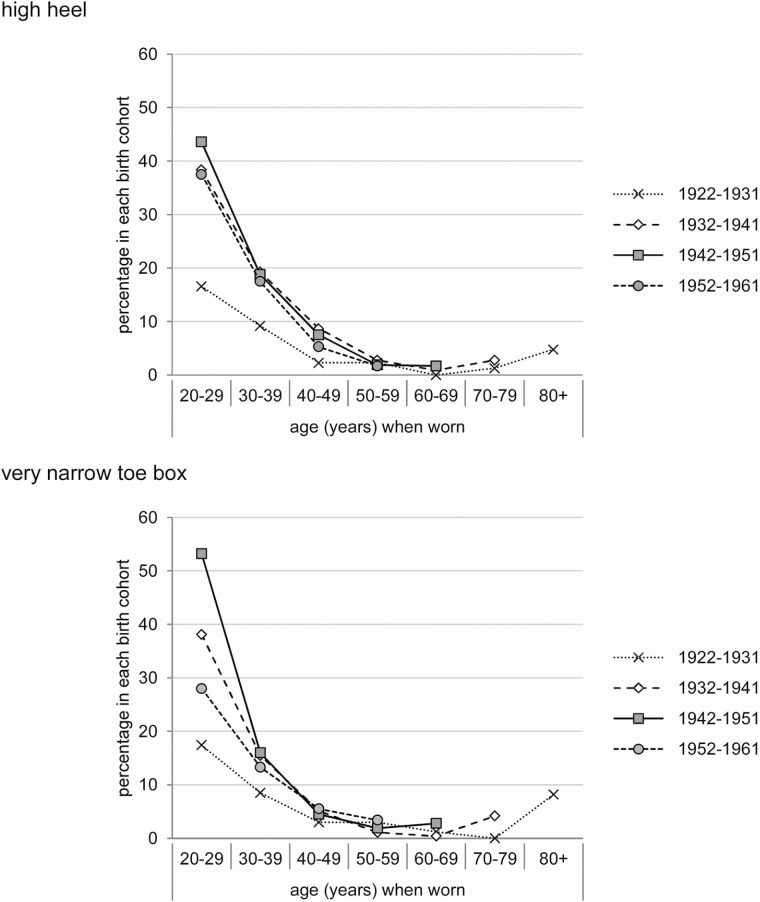
Percentage of participants who reported wearing shoes with a high heel (top) and very narrow toe box (bottom) at each decade of their life according to birth cohort.

The proportions of participants who reported wearing shoes with high heels and a narrow toe box for each decade of their life according to birth cohort are shown in Figure 2. The frequency of high heel and narrow toe box use was highest between the ages of 20 and 29 years for all birth cohorts (38% of the whole sample), and then declined across subsequent decades. A birth cohort effect was evident, with those born between 1942 and 1951 being more likely than the other cohorts to wear shoes with high heels and a narrow toe box when aged between 20 and 29 years (OR 1.55, 95% CI 1.29–1.86, *p* < .001 and OR 2.99, 95% CI 2.49–3.60, *p* < .001, respectively) and 30 and 39 years (OR 1.36, 95% CI 1.07–1.71, *p* = .009 and OR 1.43, 95% CI 1.11–1.83, *p* = 0.006, respectively). However, this birth cohort effect diminished across subsequent decades of life.

### Association Between Footwear Characteristics and Foot Pain

Heel height and toe-box shape worn between the ages of 20–29 years and 30–39 years in participants with and without foot pain in the past 12 months are shown in Table 2. There was no association between these footwear characteristics and foot pain in the past 12 months for either decade of exposure. Furthermore, there was no association with the total number of decades of exposure to high heels or a narrow toe box (data not shown). Adjusting for educational attainment did not substantially alter these associations, and similar results were obtained when adjusting for occupation instead of educational attainment (data not shown). Similar patterns were evident when these analyses were stratified by birth cohort (see Supplementary File 1).

**Table 2.** Heel Height and Toe Box Shape Worn Between the Ages of 20–29 Years and 30–39 Years in Older Women With and Without Foot Pain in the Past 12 Months

	No foot pain (*n* = 1,332)	Foot pain (*n* = 1,185)	Unadjusted		Adjusted*	
			OR (95% CI)	*p*	OR (95% CI)	*p*
Heel height						
20–29 y						
Flat	70 (6.4)	63 (6.2)	1		1	
Low	201 (18.4)	211 (20.7)	1.17 (0.79–1.73)	.441	1.15 (0.77–1.72)	.507
Medium	378 (34.6)	381 (37.4)	1.12 (0.77–1.62)	.547	1.10 (0.75–1.61)	.629
High	445 (40.7)	364 (35.7)	0.91 (0.63–1.31)	.610	0.88 (0.60–1.28)	.495
30–39 y						
Flat	100 (9.4)	104 (10.4)	1		1	
Low	298 (28.0)	319 (32.0)	1.03 (0.75–1.41)	.858	1.03 (0.74–1.42)	.873
Medium	466 (43.8)	413 (41.4)	0.85 (0.63–1.16)	.304	0.88 (0.64–1.19)	.401
High	201 (18.9)	162 (16.2)	0.78 (0.55–1.09)	.146	0.76 (0.54–1.08)	.131
Toe-box shape						
20–29 y						
Very wide	32 (2.9)	25 (2.5)	1		1	
Wide	185 (17.0)	150 (14.9)	1.04 (0.59–1.83)	.898	1.09 (0.62–1.93)	.767
Narrow	449 (41.3)	444 (44.1)	1.27 (0.74–2.17)	.392	1.34 (0.78–2.32)	.289
Very narrow	421 (38.7)	387 (38.5)	1.18 (0.69–2.02)	.556	1.24 (0.72–2.14)	.447
30–39 y						
Very wide	29 (2.8)	38 (3.8)	1		1	
Wide	267 (25.4)	277 (28.0)	0.79 (0.48–1.32)	.371	0.83 (0.49–1.39)	.826
Narrow	595 (56.6)	546 (55.2)	0.70 (0.43–1.15)	.160	0.74 (0.45–1.23)	.744
Very narrow	161 (15.3)	128 (12.9)	0.61 (0.36–1.04)	.068	0.65 (0.38–1.11)	.115

*Notes:* CI = confidence interval; OR = odds ratio.

*Adjusted for educational attainment.

### Association Between Footwear Characteristics and Hallux Valgus

Heel height and toe-box shape worn between the ages of 20–29 years and 30–39 years in participants with and without hallux valgus are shown in Table 3. There was no association between heel height and hallux valgus for either decade of exposure. However, participants with hallux valgus were more likely to have worn shoes with a narrower toe box when aged between 20–29 years and 30–39 years. Adjusting for educational attainment slightly attenuated these associations and similar results were obtained when adjusting for occupation (data not shown). When these analyses were stratified by birth cohort, similar patterns were evident (see Supplementary File 1). There was no association between the total number of decades of exposure to high heels or a narrow toe box and hallux valgus (data not shown).

**Table 3.** Heel Height and Toe Box Shape Worn Between the Ages of 20–29 Years and 30–39 Years in Older Women With and Without Hallux Valgus

	No Hallux Valgus (*n* = 1,455)	Hallux Valgus (*n* = 1,172)	Unadjusted		Adjusted*	
			OR (95% CI)	*p*	OR (95% CI)	*p*
Heel height						
20–29 years						
Flat	80 (6.5)	60 (6.5)	1		1	
Low	241 (19.5)	179 (19.2)	0.99 (0.67–1.46)	.961	0.95 (0.64–1.42)	.952
Medium	440 (35.6)	340 (36.6)	1.03 (0.72–1.48)	.872	1.03 (0.70–1.50)	.894
High	475 (38.4)	351 (36.6)	0.99 (0.69–1.42)	.936	0.98 (0.67–1.43)	.918
30–39 years						
Flat	121 (10.0)	89 (9.8)	1		1	
Low	358 (29.7)	272 (30.0)	1.03 (0.75–1.42)	.841	1.02 (0.74–1.40)	.928
Medium	518 (43.0)	380 (41.9)	1.00 (0.74–1.35)	.986	1.01 (0.74–1.38)	.946
High	208 (17.3)	165 (18.2)	1.08 (0.77–1.52)	.665	1.12 (0.79–1.58)	.542
Toe-box shape						
20–29 years						
Very wide	44 (3.6)	14 (1.5)	1		1	
Wide	212 (17.4)	132 (14.3)	1.96 (1.03–3.71)	.040	1.78 (0.93–3.38)	.080
Narrow	521 (42.7)	396 (43.0)	2.39 (1.29–4.42)	.006	2.32 (1.25–4.31)	.008
Very narrow	443 (36.3)	380 (41.2)	2.70 (1.46–5.00)	.002	2.50 (1.34–4.64)	.004
30–39 years						
Very wide	46 (3.9)	21 (2.4)	1		1	
Wide	331 (27.7)	224 (25.1)	1.48 (0.86–2.55)	.156	1.47 (0.85–2.56)	.172
Narrow	658 (55.1)	507 (56.8)	1.69 (0.99–2.87)	.052	1.69 (0.99–2.91)	.056
Very narrow	159 (13.3)	140 (15.7)	1.93 (1.10–3.39)	.022	1.96 (1.10–3.49)	.022

*Notes:* CI = confidence interval; OR = odds ratio.

*Adjusted for educational attainment.

### Population Attributable Fractions

The PAF for the association between toe-box shape and hallux valgus was calculated for two scenarios: (i) the adoption of a very wide toe box between the ages of 20 and 29 years, and (ii) the adoption of a wide or very wide toe box between the ages of 20 and 29 years. For scenario (i), the PAF was 0.408, indicating that 40.8% of cases of hallux valgus could be attributable to wearing shoes with a wide, narrow, or very narrow toe box. For scenario (ii), the PAF was 0.168, indicating that 16.8% of cases of hallux valgus could be attributable to wearing shoes with a narrow or very narrow toe box. Given the prevalence of hallux valgus in the population was 43%, these calculations indicate that (i) if women wearing shoes with a very narrow, narrow, or wide toe-box between the ages of 20 and 29 years had worn shoes with a very wide toe box, approximately 18 out of every 100 cases of hallux valgus in older women might have been prevented, and (ii) if women wearing shoes with a very narrow or narrow toe box between the ages of 20 and 29 years had worn shoes with a very wide or wide toe box, approximately 7 out of every 100 cases of hallux valgus in older women might have been prevented.

## Discussion

The findings of our population-based study suggest that shoe wearing behaviors change over time. Specifically, our results indicate that women tend to wear shoes with a lower heel and a broader toe box as they age. A similar pattern was evident across all birth cohorts and was characterized by a marked reduction in the wearing of styles of footwear with high heels and narrow toe boxes from the age of 40 onwards. Our findings are consistent with Dawson *et al.* (5), who reported a reduction in maximum heel height in shoes ‘worn for work’ between the ages of 20 and 60 in women aged 50–70 years. This change in behavior may be indicative of a life-course trajectory in which the influence and perceived importance of fashion diminishes over time and is replaced with a greater emphasis on comfort and practicality (18,25).

A birth cohort effect was observed in relation to footwear worn between the ages of 20–29 years and 30–39 years, with those born between 1942 and 1951 being more likely to wear shoes with high heels and a narrow toe box at these ages. This finding may be explained by the styles of footwear that were ‘in fashion’ during the time period when participants were aged in their 20s. The 1942–1951 birth cohorts were in their 20s in the 1960s, a decade in which stiletto heels became popular. In contrast, the 1922–1931 birth cohorts were less likely to have worn shoes with high heels and narrow toe boxes when aged between 20 and 39 years. This birth cohort was aged in their 20s in the 1940s and 1950s: a period encompassing World War II and the postwar Age of Austerity. During this period, the soles of women’s shoes were manufactured from relatively inexpensive materials such as cork, and as a consequence generally had a low heel (26).

In contrast to previous studies (12,14,15), we found no association between footwear characteristics and foot pain, although this can be largely explained by methodological differences. Previous cross-sectional studies involved the examination of *current* footwear and its association with *current* foot pain, and incorporated an assessment of the mismatch between foot size and shoe size (12,14). Our study explored the association between *previous* footwear use and foot pain in the last year, and did not involve any direct measurement of foot or shoe shape. A similar study involving recall of previous footwear use by Dufour *et al*. (15) did report an association with foot pain, although the classification of shoe characteristics was substantially different. In our study, we focused on two specific features of shoes—the height of the heel and the shape of the toe box—while Dufour *et al*. (15) collapsed 11 different shoe types into three categories (‘good’, ‘average’, and ‘poor’) and reported a protective effect of ‘good’ shoes compared with ‘average’ shoes. Interestingly, Dufour *et al*. (15) did not find any association between past use of ‘poor’ footwear and foot pain, although this may be because this category included quite disparate shoe designs (high heels, sandals, and slippers). Irrespective of differences in footwear categorization, establishing a temporal association between previous footwear use and current foot pain is inherently difficult, as foot symptoms are not stable over time (27,28). Furthermore, our foot pain question was broad and would have identified pain resulting from a diverse range of causes in addition to those related to footwear.

We found no association between hallux valgus and heel height of footwear worn between the ages of 20–29 years or 30–39 years, or between the hallux valgus and total number of decades of exposure to high heels. Dawson *et al*. (5) reported that hallux valgus was associated with a significantly lower age at which one-inch heels were first worn, although the mean age difference was small and of borderline significance (mean [*SD*] = 14.5 [2.5] vs 15.4 [2.0] years, *p* = .05). More recently, Nguyen *et al.* (16) found that older women who reported wearing shoes with a heel higher than two inches as their ‘usual’ footwear between the ages of 20 and 64 were more likely to have hallux valgus; however, this association was also of small magnitude and borderline significance (OR 1.2, 95% CI 1.0–1.5, *p* = 0.04). Taken together, these findings suggest that there is no strong association between history of wearing high heels and hallux valgus (29).

An important finding of our study is the significant association between the use of shoes with a narrow toe box and hallux valgus. Although the cause of hallux valgus is considered to be multifactorial (involving biomechanical (30) and genetic (31,32) factors), constrictive footwear may contribute to its development by holding the hallux in an abducted position, leading to soft tissue and osseous remodeling over time (29). We found a graded increase in the risk of hallux valgus with increasing narrowness of the toe box in footwear worn between 20 and 29 years of age, and to a lesser extent, at ages 30 and 39 years. This observation is suggestive of a critical period in which exposure to constrictive footwear may lead to permanent structural change. Although the prevalence of hallux valgus across the lifespan has not been examined in detail, it is uncommon in children (33) and exhibits a strong linear association with age beyond 30 years (34).

Key strengths of this study include the large, population-based sample, clearly defined evaluation of footwear characteristics and the use of a validated measure of hallux valgus. However, our findings need to be interpreted in the context of several methodological limitations. First, the accuracy of retrospective self-report of footwear use over such a long time period cannot be confirmed and is likely to be subject to recall bias. Although the footwear questions preceded the hallux valgus questions in the health survey, it is possible that women with hallux valgus may have attributed their condition to inappropriate footwear and were more likely to recall wearing constrictive footwear than those without. Second, our footwear assessment tool was limited to two key features: heel height and toe-box shape. These features were selected as they have the most plausible association with hallux valgus. However, we acknowledge that there are several other footwear characteristics that may also be of relevance to the development of foot problems (35). Finally, inferring temporal relationships between past footwear use and foot problems is inherently problematic. This is particularly the case for foot pain, the natural history of which is characterized by intermittent episodes of onset and resolution (27,28). A stronger case for temporality can perhaps be made for the association between constrictive footwear and hallux valgus, as the condition is uncommon in the 20- to 29-year age range and develops gradually. However, we cannot be certain that exposure to narrow toe-box footwear preceded the development of hallux valgus, particularly in the 30- to 39-year exposure period.

The key public health implication of our study is that avoiding constrictive footwear, particularly between the ages of 20 and 29 years, may contribute to the prevention of hallux valgus. For example, the PAF calculations indicate that approximately 18 out of every 100 cases of hallux valgus in older women might have been prevented if all women had worn shoes with a very wide toe box between the ages of 20 and 29 years. This ‘best-case’ scenario is admittedly an unrealistic goal given the limited availability of such footwear and the competing demands of fashion. However, if women wearing shoes with a very narrow or narrow toe box between the ages of 20 and 29 years had worn shoes with a very wide *or* wide toe box—a more achievable outcome—the PAFs suggest that approximately 7 out of every 100 cases of hallux valgus in older women might have been prevented. Given the high prevalence of hallux valgus in this population (33) and the economic burden associated with surgical treatment of the condition (6), this potentially represents a substantial public health benefit.

In conclusion, this study provides evidence that women tend to wear shoes with a lower heel and broader toe box as they age, but that the wearing of constrictive footwear between the ages of 20 and 39 years may be a critical period for the development of hallux valgus in later life. These findings add to the body of literature pertaining to the potentially harmful effects of footwear and suggest that advising younger women to minimize their use of constrictive shoes may have long-term benefits on foot health.

## Supplementary Material

Please visit the article online at http://gerontologist.oxfordjournals.org/ to view supplementary material.

## Funding

This work is supported by an Arthritis Research UK Programme Grant (18174) and service support through the West Midlands North CLRN.

## Conflict of Interest

The authors have no conflicts of interest to declare.

## Supplementary Material

Supplementary Data
