# Supplementary material for: Epidemiology of Shoe Wearing Patterns Over Time in Older Women: Associations With Foot Pain and Hallux Valgus
Source: J Gerontol A Biol Sci Med Sci. 2016 Feb 1;71(12):1682–7. doi: 10.1093/gerona/glw004 (PMC5106851; doi:10.1093/gerona/glw004)
Supplement: Supplementary Data [file supp_71_12_1682__index.html]

Epidemiology of Shoe Wearing Patterns Over Time in Older Women: Associations With Foot Pain and Hallux Valgus — Epidemiology of Shoe Wearing Patterns Over Time in Older Women: Associations With Foot Pain and Hallux Valgus — Supplementary Data 

# Epidemiology of Shoe Wearing Patterns Over Time in Older Women: Associations With Foot Pain and Hallux Valgus

## Supplementary Data

Data files

- Supplementary Data - Supplementary Data
