# Supplementary material for: Epidemiology of Shoe Wearing Patterns Over Time in Older Women: Associations With Foot Pain and Hallux Valgus
Source: J Gerontol A Biol Sci Med Sci. 2016 Feb 1;71(12):1682–7. doi: 10.1093/gerona/glw004 (PMC5106851; doi:10.1093/gerona/glw004)
Supplement: Supplementary Data [file supp_glw004_Supplementary_file_1.docx]

## Supplementary file 1. Associations between footwear characteristics and foot problems by birth cohort.

**Association between shape of toe-box and foot pain in the past 12 months by birth cohort.**

|  | Total sample (n=2,627) | | 1922-1931 (n=281) | | 1932-1941 (n=650) | | 1942-1951 (n=874) | | 1952-1961 (n=822) | |
| --- | --- | --- | --- | --- | --- | --- | --- | --- | --- | --- |
|  | OR (95%CI) | *P* | OR (95%CI) | *P* | OR (95%CI) | *P* | OR (95%CI) | *P* | OR (95%CI) | *P* |
| 20 to 29 years |  |  |  |  |  |  |  |  |  |  |
| Very wide | 1.00 |  | 1.00 |  | 1.00 |  | 1.00 |  | 1.00 |  |
| Wide | 1.04 (0.59 to 1.83) | 0.898 | 3.13 (0.32 to 30.50) | 0.326 | 0.76 (0.19 to 3.05) | 0.694 | 0.29 (0.08 to 1.08) | 0.065 | 1.55 (0.71 to 3.37) | 0.275 |
| Narrow | 1.27 (0.74 to 2.17) | 0.392 | 4.38 (0.47 to 40.78) | 0.194 | 0.87 (0.23 to 3.32) | 0.835 | 0.59 (0.17 to 2.05) | 0.406 | 1.47 (0.69 to 3.10) | 0.317 |
| Very narrow | 1.18 (0.69 to 2.02) | 0.556 | 5.67 (0.56 to 57.23) | 0.142 | 0.85 (0.22 to 3.26) | 0.810 | 0.52 (0.15 to 1.80) | 0.299 | 1.31 (0.61 to 2.83) | 0.487 |
| 30 to 39 years |  |  |  |  |  |  |  |  |  |  |
| Very wide | 1.00 |  | 1.00 |  | 1.00 |  | 1.00 |  | 1.00 |  |
| Wide | 0.79 (0.48 to 1.32) | 0.371 | 1.33 (0.11 to 15.82) | 0.820 | 0.68 (0.19 to 2.41) | 0.552 | 0.30 (0.08 to 1.14) | 0.076 | 1.08 (0.55 to 2.12) | 0.827 |
| Narrow | 0.70 (0.43 to 1.15) | 0.160 | 2.68 (0.24 to 30.61) | 0.427 | 0.48 (0.14 to 1.62) | 0.237 | 0.28 (0.08 to 1.01) | 0.052 | 0.84 (0.43 to 1.63) | 0.607 |
| Very narrow | 0.61 (0.36 to 1.04) | 0.068 | 3.20 (0.23 to 45.19) | 0.389 | 0.50 (0.14 to 1.82) | 0.294 | 0.23 (0.61 to 0.90) | 0.034 | 0.66 (0.31 to 1.40) | 0.278 |

**Association between shape of toe-box and hallux valgus by birth cohort.**

|  | Total sample (n=2,627) | | 1922-1931 (n=281) | | 1932-1941 (n=650) | | 1942-1951 (n=874) | | 1952-1961 (n=822) | |
| --- | --- | --- | --- | --- | --- | --- | --- | --- | --- | --- |
|  | OR (95%CI) | *P* | OR (95%CI) | *P* | OR (95%CI) | *P* | OR (95%CI) | *P* | OR (95%CI) | *P* |
| 20 to 29 years |  |  |  |  |  |  |  |  |  |  |
| Very wide | 1.00 |  | 1.00 |  | 1.00 |  | 1.00 |  | 1.00 |  |
| Wide | 1.96 (1.03 to 3.71) | 0.040 | 1.37 (0.25 to 7.54) | 0.719 | 3.12 (0.61 to 16.09) | 0.174 | 0.99 (0.27 to 3.73) | 0.993 | 2.31 (0.84 to 6.37) | 0.104 |
| Narrow | 2.39 (1.29 to 4.42) | 0.006 | 1.76 (0.34 to 9.21) | 0.505 | 3.85 (0.78 to 18.99) | 0.097 | 1.15 (0.33 to 4.03) | 0.824 | 2.89 (1.08 to 7.69) | 0.034 |
| Very narrow | 2.70 (1.46 to 5.00) | 0.002 | 3.29 (0.54 to 20.08) | 0.198 | 3.96 (0.81 to 19.61) | 0.091 | 1.55 (0.48 to 5.37) | 0.490 | 2.80 (1.03 to 7.59) | 0.043 |
| 30 to 39 years |  |  |  |  |  |  |  |  |  |  |
| Very wide | 1.00 |  | 1.00 |  | 1.00 |  | 1.00 |  | 1.00 |  |
| Wide | 1.48 (0.86 to 2.55) | 0.156 | 4.00 (0.34 to 47.50) | 0.272 | 1.57 (0.47 to 5.27) | 0.464 | 1.04 (0.32 to 3.33) | 0.952 | 1.30 (0.61 to 2.80) | 0.498 |
| Narrow | 1.69 (0.99 to 2.87) | 0.052 | 3.50 (0.31 to 39.96) | 0.313 | 1.52 (0.47 to 4.91) | 0.484 | 1.19 (0.38 to 3.68) | 0.769 | 1.37 (0.65 to 2.92) | 0.409 |
| Very narrow | 1.93 (1.10 to 3.39) | 0.022 | 3.60 (0.26 to 50.33) | 0.341 | 1.18 (0.34 to 4.08) | 0.795 | 1.55 (0.48 to 5.00) | 0.467 | 2.12 (0.93 to 4.84) | 0.073 |

**Association between heel height and foot pain in the past 12 months by birth cohort.**

|  | Total sample (n=2,627) | | 1922-1931 (n=281) | | 1932-1941 (n=650) | | 1942-1951 (n=874) | | 1952-1961 (n=822) | |
| --- | --- | --- | --- | --- | --- | --- | --- | --- | --- | --- |
|  | OR (95%CI) | *p* | OR (95%CI) | *p* | OR (95%CI) | *p* | OR (95%CI) | *p* | OR (95%CI) | *p* |
| 20 to 29 years |  |  |  |  |  |  |  |  |  |  |
| Flat | 1.00 |  | 1.00 |  | 1.00 |  | 1.00 |  | 1.00 |  |
| Low | 1.17 (0.79 to 1.73) | 0.441 | 1.00 (0.41 to 2.46) | 1.00 | 1.52 (0.64 to 3.62) | 0.348 | 0.88 (0.39 to 1.97) | 0.750 | 1.27 (0.66 to 2.45) | 0.469 |
| Medium | 1.12 (0.77 to 1.62) | 0.547 | 0.96 (0.37 to 2.50) | 0.931 | 1.55 (0.69 to 3.54) | 0.290 | 0.81 (0.37 to 1.74) | 0.584 | 1.20 (0.65 to 2.20) | 0.557 |
| High | 0.91 (0.63 to 1.31) | 0.610 | 1.00 (0.34 to 2.91) | 1.00 | 1.39 (0.62 to 3.11) | 0.418 | 0.63 (0.29 to 1.34) | 0.227 | 0.93 (0.51 to 1.71) | 0.821 |
| 30 to 39 years |  |  |  |  |  |  |  |  |  |  |
| Flat | 1.00 |  | 1.00 |  | 1.00 |  | 1.00 |  | 1.00 |  |
| Low | 1.03 (0.75 to 1.41) | 0.858 | 0.97 (0.40 to 2.34) | 0.940 | 1.40 (0.64 to 3.09) | 0.398 | 0.76 (0.41 to 1.41) | 0.386 | 1.09 (0.67 to 1.76) | 0.738 |
| Medium | 0.85 (0.63 to 1.16) | 0.304 | 1.44 (0.56 to 3.69) | 0.452 | 0.99 (0.47 to 2.08) | 0.981 | 0.64 (0.35 to 1.15) | 0.137 | 0.82 (0.51 to 1.32) | 0.415 |
| High | 0.78 (0.55 to 1.09) | 0.146 | 0.94 (0.27 to 3.34) | 0.927 | 1.00 (0.44 to 2.25) | 1.00 | 0.57 (0.30 to 1.09) | 0.089 | 0.76 (0.44 to 1.30) | 0.319 |

**Association between heel height and hallux valgus by birth cohort.**

|  | Total sample (n=2,627) | | 1922-1931 (n=281) | | 1932-1941 (n=650) | | 1942-1951 (n=874) | | 1952-1961 (n=822) | |
| --- | --- | --- | --- | --- | --- | --- | --- | --- | --- | --- |
|  | OR (95%CI) | *p* | OR (95%CI) | *p* | OR (95%CI) | *P* | OR (95%CI) | *p* | OR (95%CI) | *p* |
| 20 to 29 years |  |  |  |  |  |  |  |  |  |  |
| Flat | 1.00 |  | 1.00 |  | 1.00 |  | 1.00 |  | 1.00 |  |
| Low | 0.99 (0.67 to 1.46) | 0.961 | 0.97 (0.40 to 2.33) | 0.937 | 1.22 (0.51 to 2.89) | 0.660 | 0.82 (0.37 to 1.80) | 0.620 | 1.10 (0.54 to 2.26) | 0.792 |
| Medium | 1.03 (0.72 to 1.48) | 0.872 | 1.09 (0.42 to 2.81) | 0.859 | 1.12 (0.50 to 2.48) | 0.785 | 1.03 (0.49 to 2.17) | 0.938 | 1.34 (0.69 to 2.61) | 0.384 |
| High | 0.99 (0.69 to 1.42) | 0.936 | 0.78 (0.27 to 2.13) | 0.598 | 1.30 (0.59 to 2.89) | 0.517 | 0.87 (0.42 to 1.83) | 0.716 | 1.42 (0.73 to 2.75) | 0.303 |
| 30 to 39 years |  |  |  |  |  |  |  |  |  |  |
| Flat | 1.00 |  | 1.00 |  | 1.00 |  | 1.00 |  | 1.00 |  |
| Low | 1.03 (0.75 to 1.42) | 0.841 | 0.90 (0.37 to 2.21) | 0.822 | 0.81 (0.37 to 1.77) | 0.588 | 0.84 (0.46 to 1.55) | 0.586 | 1.24 (0.74 to 2.09) | 0.416 |
| Medium | 1.00 (0.74 to 1.35) | 0.986 | 0.84 (0.33 to 2.17) | 0.722 | 0.76 (0.36 to 1.60) | 0.465 | 0.90 (0.50 to 1.62) | 0.723 | 1.15 (0.69 to 1.91) | 0.590 |
| High | 1.08 (0.77 to 1.52) | 0.665 | 1.16 (0.31 to 4.27) | 0.826 | 0.83 (0.37 to 1.88) | 0.657 | 0.88 (0.46 to 1.66) | 0.682 | 1.48 (0.84 to 2.61) | 0.176 |
